# GABA-BZD Receptor Modulating Mechanism of *Panax quinquefolius* against 72-h Sleep Deprivation Induced Anxiety like Behavior: Possible Roles of Oxidative Stress, Mitochondrial Dysfunction and Neuroinflammation

**DOI:** 10.3389/fnins.2016.00084

**Published:** 2016-03-07

**Authors:** Priyanka Chanana, Anil Kumar

**Affiliations:** Pharmacology Division, University Institute of Pharmaceutical Sciences, UGC Centre of Advanced Study, Panjab UniversityChandigarh, India

**Keywords:** anxiety, GABA, neuroinflammation, oxidative stress, *Panax quinquefolius*, sleep deprivation

## Abstract

**Rationale:** *Panax quinquefolius* (American Ginseng) is known for its therapeutic potential against various neurological disorders, but its plausible mechanism of action still remains undeciphered. GABA (Gamma Amino Butyric Acid) plays an important role in sleep wake cycle homeostasis. Thus, there exists rationale in exploring the GABA-ergic potential of *Panax quinquefolius* as neuroprotective strategy in sleep deprivation induced secondary neurological problems.

**Objective:** The present study was designed to explore the possible GABA-ergic mechanism in the neuro-protective effect of *Panax quinquefolius* against 72-h sleep deprivation induced anxiety like behavior, oxidative stress, mitochondrial dysfunction, HPA-axis activation and neuroinflammation.

**Materials and Methods:** Male l*aca* mice were sleep deprived for 72-h by using Grid suspended over water method. *Panax quinquefolius* (American Ginseng 50, 100, and 200 mg/kg) was administered alone and in combination with GABA modulators (GABA Cl^−^ channel inhibitor, GABA-benzodiazepine receptor inhibitor and GABAA agonist) for 8 days, starting 5 days prior to 72-h sleep deprivation period. Various behavioral (locomotor activity, mirror chamber test), biochemical (lipid peroxidation, reduced glutathione, catalase, nitrite levels), mitochondrial complexes, neuroinflammation marker (Tumor Necrosis Factor, TNF-alpha), serum corticosterone, and histopathological sections of brains were assessed.

**Results:** Seventy two hours sleep deprivation significantly impaired locomotor activity, caused anxiety-like behavior, conditions of oxidative stress, alterations in mitochondrial enzyme complex activities, raised serum corticosterone levels, brain TNFα levels and led to neuroinflammation like signs in discrete brain areas as compared to naive group. *Panax quinquefolius* (100 and 200 mg/kg) treatment restored the behavioral, biochemical, mitochondrial, molecular and histopathological alterations. Pre-treatment of GABA Cl^−^ channel inhibitor as well as GABA-benzodiazepine receptor inhibitor, significantly reversed the protective effect of *P. quinquefolius* (100 mg/kg) in 72-h sleep deprived animals (*P* < 0.05). However, pretreatment with GABAA agonist, potentiated *Panax quinquefolius's* protective effect which was significant as compared to their effect *per se* (*p* < 0.05).

**Conclusion:** GABA-ergic mechanism could be involved in the neuroprotective effect of *P*.quinquefolius against sleep deprivation induced anxiety-like behavior, oxidative stress, mitochondrial dysfunction, HPA axis activation and neuroinflammation.

## Introduction

GABA (Gamma-amino-butyric-acid) is the cardinal inhibitory neurotransmitter of the central nervous system (CNS) (Parades and Agmo, 1992). It exerts its effects via three types of GABA receptors viz ionotropic GABA_A_and GABAc (both coupled to Cl^−^ ionophore), and metabotropic GABA_*B*_ receptors (which are coupled to Ca^2+^ and K^+^ ion channels and function via the involvement of a second messenger) (Barnard et al., 1998; Bowery, 2006). The GABA_A_ receptors are predominantly expressed in the central nervous system while GABA_C_ receptors are primarily expressed in retina and optical layers of the superior colliculi (Bloom and Iversen, 1971; McCabe and Wamsley, 1986). The GABA_A_ receptor mediated Cl^−^ channel conductance is responsible for the GABA-ergic inhibitory neurotransmission in the CNS (Turner and Whittle, 1983).

GABA is also an important mediator of the sleep/wake flip-flop cycle and plays pivotal role in maintenance of sleep homeostasis. The sleep promoting VLPO (Ventero-lateral preoptic nuclei) neurons of the anterior hypothalamus are responsible for quietening the ascending monoaminergic arousal system mediate their response via the release of two inhibitory neurotransmitters GABA and galanin. Thus, GABAergic neurons in the VLPO and adjacent BF (basal forebrain) play a critical role in the induction and maintenance of sleep (Gallopin et al., 2000; Manns et al., 2003; Jones, 2005).

Sleep deprivation [SD] has been reported to alter the content of the GABA, suggesting that the GABA-ergic mechanism play a critical role in sleep deprivation-induced behavioral alterations and oxidative stress (Modirrousta et al., 2007). The secondary detrimental manifestations associated with sleep deprivation include anxiety like behavior, cognitive deficits, oxidative stress (via accumulation of reactive oxygen species (ROS), mitochondrial dysfunction, neuroinflammation etc. that have all been individually responsible for affecting the GABA-ergic transmission. Not only this, the various allosteric binding sites of GABAA receptor have also served as potential therapeutic targets for the management of SD induces anxiety like problems (Modirrousta et al., 2007; Tadavarty et al., 2011). In addition to the mitochondrial electron transport chain, oxidative dysregulation and accretion of ROS as a consequence of SD may also be attributed to extramitochondrial sources such as nicotinamide adenosine dinucleotide phosphate oxidases (NOX) (Nair et al., 2011). The NOX are membrane bound enzyme complexes that transfer electrons across biological membranes to produce highly reactive superoxide ion and serve potent inducers of oxidative dysregulation. Within the family of seven different NOX isotypes, NOX2 is a prototype NADPH oxidase that plays cardinal role in ROS generation in the CNS and thus leads to neuronal degeneration via activation of oxidative stress cascades contributing to extensive behavioral and pathological conditions in several neuropsychiatric and neurodegenerative diseases like anxiety, psychosis, schizophrenia, Alzheimer's, Parkinson's disease etc. (Bedard and Krause, 2007; Behrens et al., 2007; Schiavone et al., 2009, 2012; Sorce and Krause, 2009).

The link between increased corticosterone levels and GABA mediated inhibitory potentials has also been explored and it has been inferred that increased levels of corticosterone due to stress slowly but persistently leads to inhibition of the GABA-ergic tone (Verkuyl et al., 2005). Furthermore, elevated levels of tumor necrosis factor (TNF-α) have also been reported to deplete inhibitory synaptic strength. In response to TNF-α binding to the neuronal TNFR1, there has been accounted not only a down-regulation of GABA-ergic signaling but a decline in the cell-surface levels of GABA_A_ receptors as well (Pribiag and Stellwagen, 2013). With the above background it is speculated that development of secondary neurological disorders as a consequence of SD can be attributed to a complex interplay between oxidative stress, mitochondrial dysfunction, neuroinflammatory responses and HPA-axis activation, all accusing detrimental effects to the GABA-ergic inhibitory signaling. Therefore, GABA-ergic signaling purports to be a crucial link between sleep homeostasis, SD and development of secondary neurological comorbidities associated with SD.

*Panax quinquefolius* (American ginseng) has served as a valuable ingredient of the traditional medicine and this may be attributed due to its array of beneficial effects produced on the CNS (Radad et al., 2011). The CNS disorders benefitted with *P. quinquefolius* treatment are found to be not only confined to the traditional target diseases such as stress, anxiety and ischemia but also expanded into progressive neurological and psychiatric disorders including Alzheimer's disease (AD), Parkinson's disease (PD), Huntington's Disease and attention deficit hyperactivity disorder (ADHD) (Kim et al., 2013). The neuroprotective aspects of *Panax quinquefolius* may be accredited to its pharmacologically active constituents named ginsenosides (mainly Rb1 and Rg1) which possess antioxidant, anti-neuroinflammatory properties as well as possess potential of modulating neurotransmitters release, synaptic plasticity and neurogenesis (Nakaya et al., 2004; Park et al., 2009; Radad et al., 2011). *P. quinquefolius* has also demonstrated to possess GABA modulating effects; literature has reported that ginsenosides regulate GABA-ergic neurotransmission via modulating the ligand binding capacities at the GABA_A_ receptor or by directly increasing the Cl^−^ channel activity by interacting with γ_2_ subunit (Kim et al., 2001; Choi S. E. et al., 2003; Lee et al., 2013). But till date the full potential of *P. quinquefolius* remains untapped in leiu of its protective effects and plausible mechanisms in SD induced secondary neurological comorbidities. Therefore, with the above mentioned backdrop, the present study had been designed with aim to investige the possible interaction of *P. quinquefolius* with GABA-ergic modulators in SD induced anxiety like behavior, conditions of oxidative stress, mitochondrial dysfunction, HPA-axis activation and neuroinflammation in mice.

## Materials and methods

### Animals

Male *laca* mice (22–30 g) bred in Central Animal House (CAH) facility of the Punjab University, Chandigarh, India were used. The animals were housed under standard laboratory conditions and maintained under natural light-dark cycles. The animals were acclimatized to laboratory conditions before actual start of the experiment. All the experiments were carried out between 09:00 and 17:00 h. The experimental protocol was approved by Institutional Animal Ethics Committee (IAEC) of Panjab University [Protocol no. IEAC/PU/282/UIPS-11, dated 5/02/2013] and carried out in accordance with the guidelines of Committee for the Purpose of Control and Supervision of Experimentation on Animals (CPCSEA), Government of India and Indian National Science Academy Guidelines for the use and care of experimental animals.

### Sleep deprivation

Animals were sleep-deprived for a total duration of 72-h by using modified grid suspended over water method developed by Shinomiya et al. (2003). Briefly, animals in a group of six to eight were sleep deprived by placing on a grid floor (29 × 15 × 7 cm) inside polyacrylic cages (38 × 23 × 10 cm), filled with water upto 1 cm below the grid surface for 72-h. The stainless steel rods of the grid (3 mm wide) were set 2-cm apart from each other. Food and water were provided *ad libitum*. As soon as the animal tried to sleep, it lost its muscle tone and thereby grip over the grid, which made it fall in water and thus awakened the animal.

### Drugs and treatment schedule

Animals were divided into different groups consisting of 10–12 animals in each group based on their treatment paradigms. Table 1 demonstrates the various treatment groups of the study.

**Table 1:** **Treatment groups**.

**S. NO**.	**Treatment Group (mg/kg)**	**Treatment**
1.	Naïve	Animals not subjected sleep deprivation, vehicle treated
2.	Control (SD-72 h)	72-h sleep deprived; vehicle treated
3.	GIN (50)	*P. quinquefolium* (50 mg/kg) administered for 8 days, started 5 days before 72-h sleep deprivation
4.	GIN (100)	*P. quinquefolium* (100 mg/kg) administered for 8 days, started 5 days before 72-h sleep deprivation
5.	GIN (200)	*P. quinquefolium* (200 mg/kg) administered for 8 days, started 5 days before 72-h sleep deprivation
6.	PTX (0.5)	Picrotoxin (0.5 mg/kg) administered for 8 days started 5 days before 72-h sleep deprivation
7.	PTX (0.5) + GIN (100)	Picrotoxin (0.5 mg/kg) pretreatment followed by *P. quinquefolium* (American Ginseng 100 mg/kg) administered for 8 days, started 5 days before 72 h sleep deprivation
8.	FLU (0.5)	Flumazenil (0.5 mg/kg) administered for 8 days started 5 days before 72-h sleep deprivation
9.	FLU (0.5) + GIN (100)	Flumazenil (0.5 mg/kg) pretreatment followed by *P. quinquefolium* (American Ginseng 100 mg/kg) administered for 8 days, started 5 days before 72 h sleep deprivation
10.	MUS (0.05)	Muscimol (0.05 mg/kg) administered for 8 days started 5 days before 72-h sleep deprivation
11.	MUS (0.05) + GIN (100)	Muscimol (0.05 mg/kg) pretreatment followed by *P. quinquefolium* (American ginseng 100 mg/kg) administered for 8 days, started 5 days before 72 h sleep deprivation

*SD-72, Sleep deprivation of 72-h; GIN, Panax quinquefolius (American ginseng); PTX, Picrotoxin; FLU, Flumazenil; MUS, Muscimol*.

**Figure 1:**
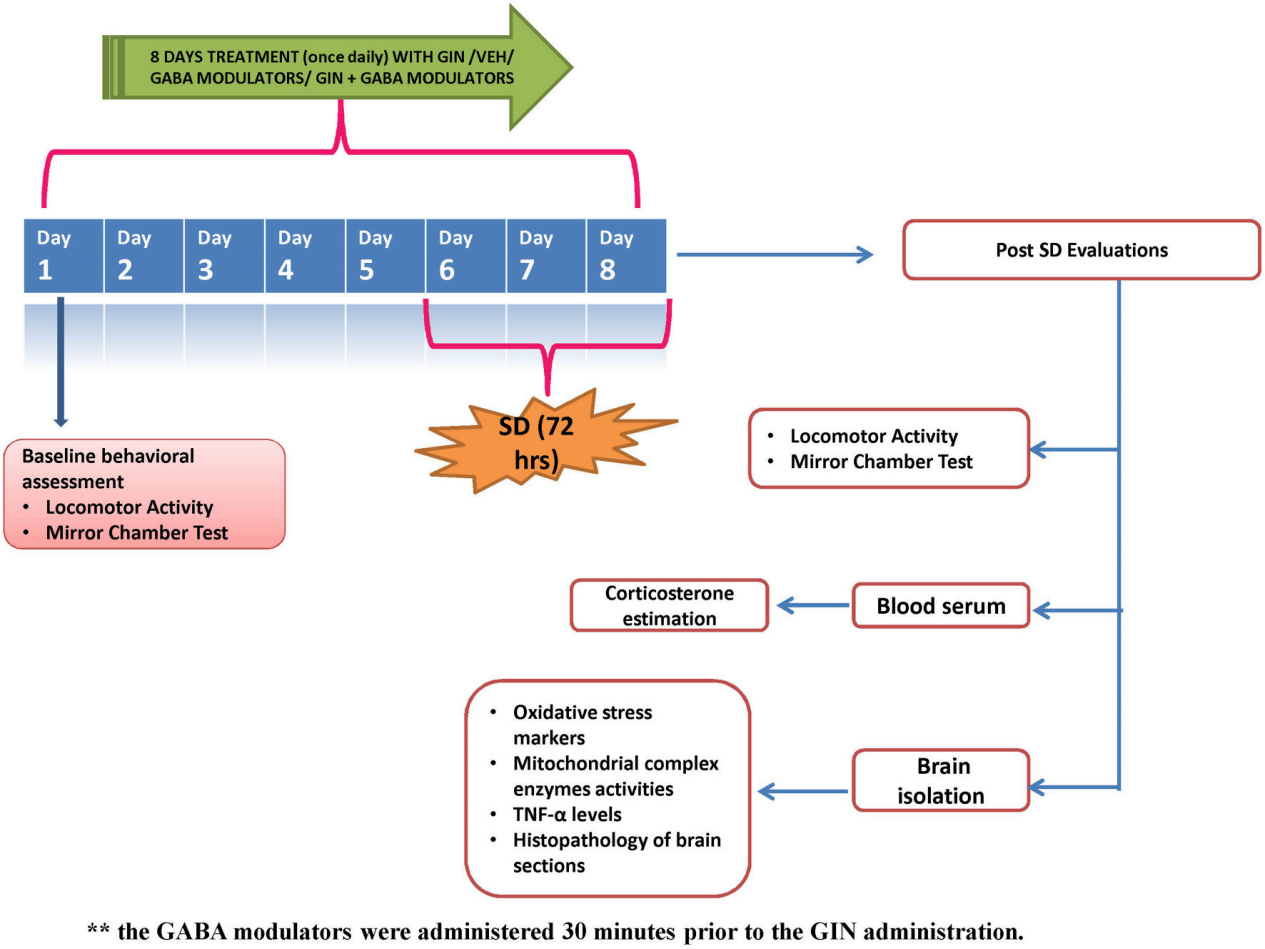
**Figure describes the experimental design adopted during the 8 day protocol**. GIN: *Panax quinquefolius* (American ginseng), SD, Sleep Deprivation of 72-h; Veh, vehicle treatment.

*American ginseng (Panax quinquefolius*, family: *Arileacea)* (Sigma-Aldrich, St. Louis, MO, USA) was suspended in 0.5% (w/v) sodium carboxy-methyl cellulose (CMC) solution and administered per oral in a constant volume of 1 ml per 100 g of bodyweight. GABA modulators (picrtoxin, flumazenil) were dissolved in 0.1% dimethylsulfoxide, while muscimol was dissolved in distilled water. The GABA modulators were administered as pre-treatments (30 min before *P. quinquefolius* administration). Each group received treatment for a total period of 8 days, starting 5 days prior to 72-h SD. Data of *per se* groups for all the treatments (where the drug would have been given to naïve animals to see their innate effects in non-challenged animals) was not shown due to ethical constraints and to minimize the use of experimental animals. The entire study protocol design has been depicted in Figure 1.

### Analysis of ginsenosides in *Panax quinquefolius*

**Figure 2:**
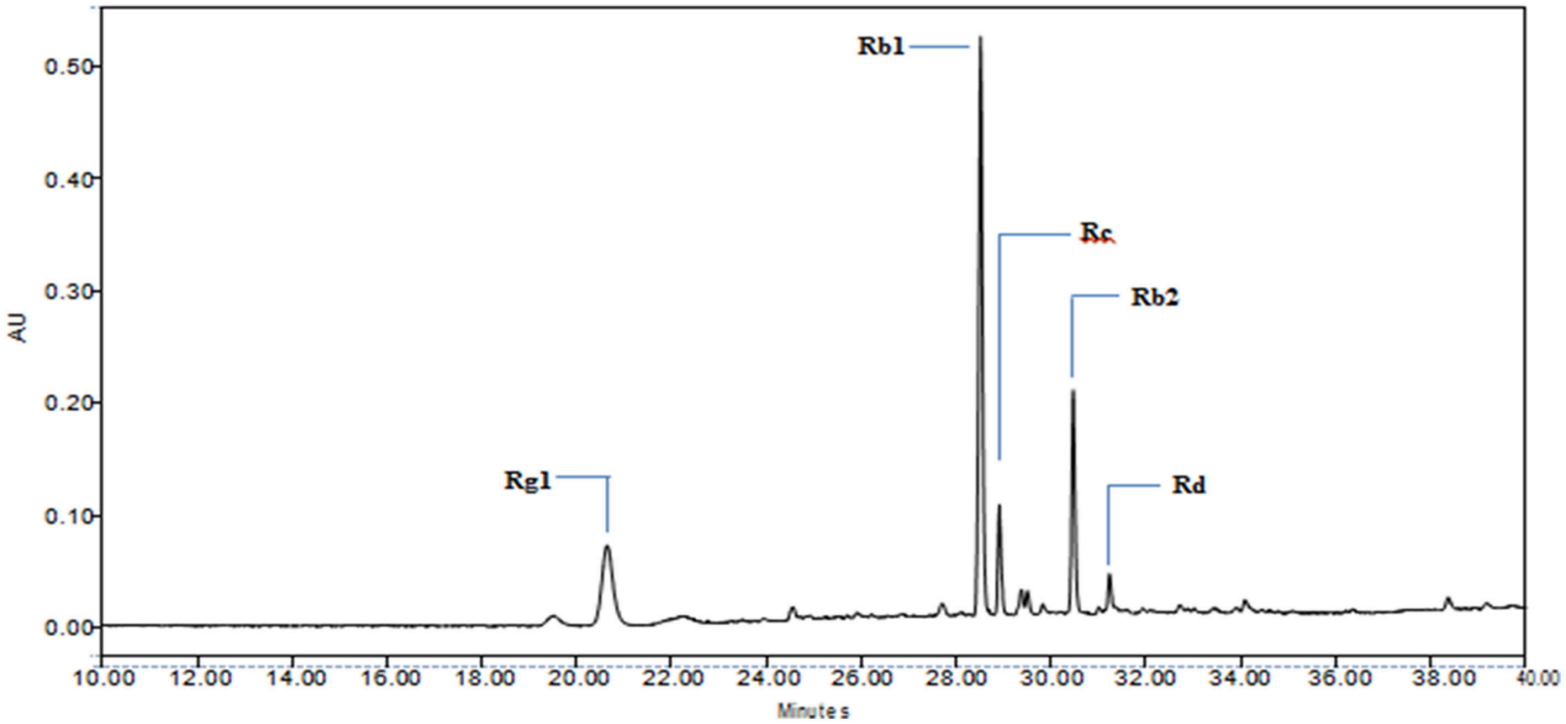
**HPLC fingerprint of *Panax quinquefolius* (American ginseng)**.

A gradient HPLC method was employed for the qualitative analysis of ginsenosides in the *P. quinquefolius* (American ginseng). The American ginseng extract as well as standards for ginsenosides (the standard ginsenosides Rb1, Rg1, Rc, Rb2, and Rd Sigma-Aldrich, USA) were dissolved in HPLC grade methanol (90:10). The HPLC system used was a Waters 2695 separation module (Waters, Milford, MA, USA) equipped with a pump, auto-injector and Waters PDA detector, all controlled by Empower software. The stationary phase used was a Thermo Fischer Symmetry C18 column (5 μm, 4.6 mm ID × 250 mm, Thermo Fischer, USA) thermostated at 25°C, the corresponding wavelength selected was 200 nm. The mobile phases consisted of (A) acetonitrile (Sigma-Aldrich, USA) and (B) HPLC grade water (Merck, USA) which were run across a gradient (gradient specifications as mentioned in Table 2) at a flow rate of 0.80 mL/min. Figure 2 shows qualitative chromatogram of different ginsenosides present in *Panax quinquefolius*.

**Table 2:** **Gadient conditions for HPLC**.

**Time (mins)**	**Mobile phase (A) (%)**	**Mobile phase (B) (%)**
0	20	80
15	20	80
18	22	78
30	46	54
40	55	45
45	100	0
50	20	80

### Behavioral assessments

#### Measurement of locomotor activity

The locomotor activity was recorded by using an actophotometer (IMCORP, Ambala, India). The instrument consisted of a closed arena equipped with 12 infrared light-sensitive photocells in two rows (six in each row), at a distance of 3 and 9 cm respectively. Before locomotor task, each animal was placed in the activity chamber for 3 min for habituation and exploration. The actual locomotor activity was then recorded for a period of 5 min. The photo beams in actophotometer, interrupted by the animal's motion were taken as the measure of movements (ambulation and rearing). Total activity (ambulation + rearing counts) was recorded and expressed in terms of total photo beam counts cut for 5 min per animal (Kalonia and Kumar, 2007).

#### Assessment of anxiety like behavior using mirrored chamber test (MCT)

The mirror chamber was a structured wooden chamber (40 × 40 × 30.5 cm) with a mirror cube of slightly lesser dimensions, kept inside the wooden chamber. At the beginning of the test, each animal was placed at the distal corner of the mirror chamber. After habituation and exploration period of initial 3 min, the test session was recorded. During the 5 min test session, the following parameters were noted (a) Latency to enter the mirror cube inside the wooden chamber, (b) Number of entries into the mirror cube (c) Total time spent in the mirror cube. An anxiogenic response was defined by increased latency to enter mirror cube, as well as decreased number of entries and reduced time spent in the mirror cube (Kalonia and Kumar, 2007).

### Biochemical tests

#### Dissection and homogenization

Thereupon, after the last behavioral test, animals were randomized into distinct sets; the first set was used for the biochemical assays (*n* = 5/group). For biochemical analysis, animals were sacrificed by decapitation. Whole brain from each animal was put over ice and weighed. 10% (w/v) tissue homogenates were prepared in 0.1 M phosphate buffer (*pH* 7.4). Following which the homogenates were centrifuged at 10,000 × g for 15 min and aliquots of supernatants were separated and used for biochemical estimations.

#### Lipid peroxidation assay

The quantitative measurement of lipid peroxidation in the whole brain was measured according to the method of Wills (1966). The amount of malondialdehyde (MDA) concentration formed was measured by the reaction with thiobarbituric acid at 532 nm using Perkin Elmer lambda 20 UV-vis spectrophotometer (Norwalk, CT, USA). The results were expressed as nano moles of MDA per milligram protein using the molar extinction coefficient of chromophore (1.56 × 10 M/cm) and represented as percentage of naive.

#### Estimation of reduced glutathione

Reduced glutathione (GSH) was estimated according to the method described by Ellman (1959). 1.0 mL of homogenate was precipitated using 1.0 mL of 4% sulfosalicylic acid by keeping the mixture at 4°C for 1 h and the samples were immediately centrifuged at 1200 g for 15 min at 4°C. The assay mixture contained 0.1 mL of supernatant, 2.7 mL of phosphate buffer (*p*H 8.0) and 0.2 mL of 0.01 M-dithiobisnitrobenzoic acid. The yellow color developed was read immediately at 412 nm using spectrophotometer (Perkin Elmer lambda 20 UV-Vis spectrophotometer (Norwalk, CT, USA). The results were expressed as nano moles of GSH per milligram of protein and represented as percentage of naive.

#### Estimation of nitrite levels

The accumulation of nitrite in the supernatant (an indicator of the production of nitric oxide (NO load), was determined with a colorimetric assay using Greiss reagent (0.1% N- (1-napthyl) ethylene diaminedihydrochloride, 1% sulfanilamide and 2.5% phosphoric acid) as per the method reported by Green et al. (1982). Equal volumes of supernatant and Greiss reagent were mixed, incubated for 10 min at room temperature and the absorbance of reaction mixture was read at 540 nm using a spectrophotometer (Perkin Elmer lambda 20 UV-Visible spectrophotometer, Norwalk, CT, USA). The concentration of nitrite in the supernatant was determined from a standard curve. The results were expressed as mg/ml and represented as percentage of naïve.

#### Estimation of catalase

Catalase activity was assayed by the method reported by Luck (1965), wherein the breakdown of hydrogen peroxide (H_2_O_2_) was measured at 240 nm. Briefly, the assay mixture consisted of 3 mL of H_2_O_2_ phosphate buffer (1.25 × 10^−2^M H_2_O_2_) and 0.05 mL of supernatant of tissue homogenate (10%). The change in absorbance was recorded at 240 nm using UV-Vis spectrophotometer (Perkin Elmer lambda 20 spectrophotometer (Norwalk, CT, USA). The results were expressed as micromole H_2_O_2_ decomposed per milligram of protein/min and represented as percentage of naive.

#### Superoxide dismutase activity

Superoxide dismutase (SOD) activity was assayed by the method of Kono (1978). According to the method, reduction of nitro blue tetrazolium (NBT) was inhibited by the SOD and the change in absorbance was recorded for 2 min at 30 s intervals by measuring absorbance at 560 nm using Perkin Elmer Lambda 20 spectrophotometer. The results were expressed as units/mg protein and represented as percentage of naive.

#### Protein estimation

The protein content was measured according to Biuret method described by Lowry et al. (1951) using bovine serum albumin as the standard.

#### Mitochondrial enzyme complex estimations

The brain samples of second set of animals were used for evaluation of mitochondrial enzyme complex activities as described by Berman and Hastings (1999). The whole brain was homogenized in isolation buffer (215 mM mannitol, 75 mM sucrose, 0.1% BSA, 20 mM HEPES, 1 mM EGTA, and *p*H 7.2). Homogenates were centrifuged at 13,000 g for 5 min at 4°C. Pellets were re-suspended in isolation buffer with ethylene glycol tetra acetic acid (EGTA) and spun again at 13,000 g at 4°C for 5 min. The resulting supernatants were transferred to new tubes and topped off with isolation buffer with EGTA and again spun at 13,000 g at 4°C for 10 min. Pellets containing pure mitochondria were re-suspended in isolation buffer without EGTA.

#### Nicotinamide adenine dinucleotide (NADH) dehydrogenase activity

Complex-I was measured spectrophotometrically by the method mentioned by King and Howard (1967). The method involved catalytic oxidation of NADH to NAD^+^ with subsequent reduction in cytochrome *c*. The reaction mixture contained 0.2 M glycyl glycine buffer *p*H 8.5, 6 mM NADH in 2 mM glycyl glycine buffer and 10.5 mM cytochrome *c*. The reaction had been initiated by addition of requisite amount of solubilised mitochondrial sample to reaction mixture, followed by record of absorbance change at 550 nm for 2 min and the results were expressed nano moles of NADH oxidized per minute per milligram protein and represented as percentage of naïve.

#### Succinate dehydrogenase (SDH) activity

Complex-II/SDH was measured spectrophotometrically according to method reported by King (1967). The method involved oxidation of succinate by an artificial electron acceptor, potassium ferricyanide. The reaction mixture contained 0.2 M phosphate buffer (*p*H 7.8), 1% BSA, 0.6 M succinic acid, and 0.03 M potassium ferricyanide. The reaction was initiated by the addition of mitochondrial sample and absorbance change was read at 420 nm for 2 min. The results were expressed as nano moles of SDH per milligram of protein and represented as percentage of naïve.

#### 3-(4,5-dimethylthiazol-2-yl)-2,5-diphenyltetrazolium (MTT) assay

Also known as mitochondrial redox activity assay, this method was based on the *in vitro* studies to evaluate the mitochondrial redox activity via the conversion of MTT tetrazolium salt to formazan crystals by mitochondrial respiratory chain reactions in isolated mitochondria by the method of Liu et al. (1997). The blue formazan crystals were solubilised with dimethylsulphoxide and corresponding absorbance was measured by an ELISA reader at 580 nm filter. The results were expressed number of viable cells per well and represented as percentage of naïve.

#### Cytochrome oxidase activity

Cytochrome oxidase activity was assayed in brain mitochondria according to the method developed by Sottocasa et al. (1967). The assay mixture contained 0.3 mM reduced cytochrome C in 75 mM phosphate buffer. The reaction was started by the addition of solubilized mitochondrial sample and the changes in absorbance were recorded at 550 nm for 2 min (at intervals of 30 s each) and the results represented as percentage of naive.

#### Estimation of serum corticosterone [CORT]

##### Preparation of serum

Blood collected in the test tube was allowed to clot at room temperature. The tubes were then centrifuged at 2000 rpm for 10 min, and the straw-colored serum was separated and stored frozen at −20°C.

Serum corticosterone was estimated as per the modified method of Silber et al. (1958). The 0.2 ml of serum was treated with 0.2 ml of freshly prepared chloroform/methanol mixture (2:1 v/v), followed by 3 ml of chloroform instead of dichloromethane used in the procedure described by Siber and his co-workers. The step of treatment of petroleum ether was also omitted. The samples were vortexed for 30 s and centrifuged at 2000 rpm for 10 min. The chloroform layer was carefully removed with the help of a long 16-gauge needled syringe and was transferred to a fresh tube. The chloroform extract was then treated with 0.1 N NaOH by vortexing rapidly, and corresponding NaOH layer was rapidly removed. The sample was then treated with 3 ml of 30 N H_2_SO_4_ and vortexed vigorously. After phase separation, chloroform layer on top was removed using a syringe as described above and discarded. The tubes containing H_2_SO_4_ were kept in dark for 30–60 min and thereafter fluorescence measurements carried out in fluorescence spectrophotometer (F-2500 Hitachi, Japan) with excitation and emission wavelength set at 472 and 523.2 nm, respectively. The standard curve depicting the fluorescence yield vs. corticosterone concentration was used for result analysis.

#### Estimation of TNF-α activity

The brain of first set of animals was used for the estimation of TNF- α levels in mice brain. The quantification of cytokine TNF-α was performed as per the instructions specified in BD Biosciences immunoassay kit (BD opt EIA by BD Bioscience, San Jones, CA, USA) The mice TNF-α immunoassay was 4.5 h solid phase ELISA which employed sandwich enzyme immune assay technique. Monoclonal antibody specific for mice TNF-α had been pre-coated in the microplate. When standards, control, and samples were pipetted into the wells, any mice-TNF-α present was bound by the immobilized antibody. After washing away any unbound substance an enzyme linked polyclonal antibody specific for mice TNF-α was added to the wells. Following a wash to remove any unbound antibody-enzyme reagent, a substrate solution was added to the wells. The enzyme reaction yielded a blue product that turned yellow when the stop solution was added. The intensity of the color measured (using a microtiter plate reader read at 450 nm) was found to be in proportion to the amount of mice TNF-α bound in the initial steps. The sample values were then read off the standard curve.

### Histopathological analysis by hematoxylin and eosin staining (H and E staining)

#### Tissue sections preparation

Third set of animals were scarified immediately after last behavioral test and perfused transcardially via the ascending aorta with cold phosphate buffered saline (0.1 M, *p*H 7.4). The whole brain was dissected out and fixed overnight at 4°C in the same buffer containing 10% (v/v) paraformaldehyde. The brain was then washed with 0.1 M PBS (*p*H 7.4) for 1 h, dehydrated in alcohol, and then embedded in paraffin wax. Serial coronal sections (5–10 μm thickness) of whole brain were then obtained using a freezing microtome.

#### Haematoxylin and eosin (H&E) staining

The brain sections (5–10 μm) thick were de-waxed and stained with haematoxylin and eosin. Briefly, sections were immersed in the filtered haematoxylin solution for 1 min followed by rinsing with tap water. Then, the sections were immersed in eosin stain for 1–2 min and rinsed thoroughly with tap water followed by dehydration in ascending alcohol solutions (50, 70, 80, 95, 97, and 100% alcoholic solutions). Now the sections were mounted on labeled slides. The stained sections were viewed under a binocular microscope (Nikon Eclipse 80i, Nikon Instruments Inc., USA) at 40 × targeting the thalamo-cortical regions of the brain for the presence of morphological changes and thereby photographed. The particular encephalic areas were analyzed for the presence and density of pyknotic nuclei. The number of such pyknotic nuclei per square pixel were analyzed using computer based image analysis (Image J 1.42q, NIH, USA).

#### Statistical analysis

All statistical procedures were carried out using sigma stat Graph Pad Prism (Graph Pad prism Software, version 5, San Diego, CA). All the values were expressed as mean ± SEM. The data was analyzed using analysis of variance (ANOVA) followed by *Post-hoc* evaluation utilizing the Tukey's test. In all the tests, the criterion for statistical significance was *p* < 0.05.

## Results

### Effects of *Panax quinquefolius* (GIN) treatment and its regulation by GABA modulators on behavioral test paradigms (locomotor activity and mirror chamber test performance) in 72-h sleep deprived animals

Seventy two hours of sleep deprivation significantly impaired locomotor activity as well as precipitated anxiety like behavior [as depicted by increased time latency to enter the mirror cube, decreased number of entries as well as depleted duration spent in mirror cube] as compared to the naive group (*p* < 0.05). *Panax quinquefolius (GIN)* treatment (100 and 200 mg/kg) for 8 days significantly improved locomotor activity as well as exhibited antianxiety like effect as compared to the control group (72-h SD) group (*p* < 0.05) (Tables 3, 4 respectively). However, *Panax quinquefolius (GIN)* 50 mg/kg did not produce any significant improvement in locomotor activity as well as anxiety like behavior as compared to 72-h sleep deprived control group. The effect of *Panax quinquefolius (GIN)* was further found to be regulated by GABA modulators. Pre-treatment of flumazenil (0.5 mg/kg) [GABA-BZD receptor antagonist] and or picrotoxin (0.5 mg/kg) [GABA chloride channel blocker] with *Panax quinquefolius (GIN)* (100 mg/kg) for 8 days, significantly reversed the protective effect of *Panax quinquefolius (GIN)* in 72-h sleep deprived animals (*p* < 0.05) as compared to their individual effects. However, muscimol [GABA_*A*_ selective agonist] (0.05 mg/kg) pretreatment with *Panax quinquefolius (GIN)* (100 mg/kg) for 8 days significantly potentiated their protective effect which was significant as compared to their effects *per se* (*p* < 0.05) (Tables 3, 4).

**Table 3:** **Effect of *Panax quinquefolius (GIN)* and its interaction with GABA modulators on locomotor activity in 72-h sleep deprived animals**.

**S. No**.	**Treatment group (mg/kg)**	**Total activity day 8 (Counts/5 min)**
1	Naïve	204.00, 2.75
2	Control (SD-72 h)	63.428, 5.68^a^
3	GIN (50)	71.66, 6.36^a^
4	GIN (100)	119.875, 8.87^b^^,^^c^
5	GIN (200)	135.67, 5.62^b^^,^^c^
6	PTX (0.5)	64.42, 5.50^a^
7	PTX (0.5) + GIN (100)	78.0, 2.65^a^^,^^d^
8	FLU (0.5)	66.21, 3.41^a^
9	FLU (0.5) + GIN (100)	81.33, 4.11^a^^,^^d^
10	MUS (0.05)	70.60, 4.61^a^
11	MUS (0.05) + GIN (100)	195.21, 3.97^b^^,^^d^^,^^e^

*Values are expressed as mean ± SEM*.

a *p < 0.05 as compared with naive*,

b *p < 0.05 as compared with control (SD-72)*,

c *p < 0.05 as compared with GIN (50 mg/kg)*,

d *p < 0.05 as compared with GIN (100 mg/kg)*,

e *p < 0.05 as compared with MUS (0.05 mg/kg)*.

*(One-way ANOVA followed by Tukey's test). SD-72, Sleep deprivation of 72-h; GIN, Panax quinquefolius (American ginseng); PTX, Picrotoxin; FLU, Flumazenil; MUS, Muscimol*.

**Table 4:** **ffect of *Panax quinquefolius (GIN)* on anxiety like behavior as represented by the Mirror Chamber Test performance and its interaction with GABA modulators in 72-h sleep deprived animals**.

**S. No**	**Treatment Groups (mg/kg)**	**Latency to enter mirror cube (seconds)**	**Number of entries in mirror cube**	**Time spent in mirror cube (seconds)**
1	Naïve	55.41, 7.54	8.42, 1.23	89.13, 6.25
2	Control (SD-72 h)	174.40, 9.66^a^	1.27, 0.61^a^	24.08, 0.75^a^
3	GIN (50)	160.27, 8.91^a^	2.35, 0.42^a^	32.13, 4.43^a^
4	GIN (100)	99.56, 4.02^b^^,^^c^	5.62, 0.3^b^^,^^c^	53.34, 4.77^b^^,^^c^
5	GIN (200)	73.14, 6.04^b^^,^^c^	6.01, 0.4^b^^,^^c^	67.42, 5.14^b^^,^^c^
6	PTX (0.5)	176.62, 7.13^a^	1.39, 0.44^a^	26.14, 1.92^a^
7	PTX (0.5) + GIN (100)	161.26, 5.61^a^^,^^d^	1.97, 0.47^a^^,^^d^	31.43, 3.12^a^^,^^d^
8	FLU (0.5)	178.45, 5.01^a^	1.51, 0.44^a^	27.43, 2.12^a^
9	FLU (0.5) + GIN (100)	157.51, 6.64^a^^,^^d^	2.22, 0.88^a^^,^^d^	34.17, 3.22^a^^,^^d^
10	MUS (0.05)	165.23, 3.11^a^	2.01, 0.81^a^	30.71, 1.46^a^
11	MUS (0.05) + GIN (100)	67.43, 1.79^b^^,^^d^^,^^e^	9.62, 1.1^b^^,^^d^^,^^e^	82.34, 4.04^b^^,^^d^^,^^e^

*Values are expressed as mean ± SEM*.

a *p < 0.05 as compared with naive*,

b *p < 0.05 as compared with control (SD-72)*,

c *p < 0.05 as compared with GIN (50 mg/kg)*,

d *p < 0.05 as compared with GIN (100 mg/kg)*,

e *p < 0.05 as compared with MUS (0.05 mg/kg)*.

*(One-way ANOVA followed by Tukey's test). SD-72, Sleep deprivation of 72-h; GIN, Panax quinquefolius (American ginseng); PTX, Picrotoxin; FLU, Flumazenil; MUS, Muscimol*.

### Effects of *Panax quinquefolius* (GIN) treatment and its modulation by GABA modulators on sleep deprivation induced oxidative stress

The results depicted in Table 5 signify that 72-h sleep deprivation significantly caused oxidative stress like conditions as evidenced by rise in lipid peroxidation, nitrite concentration, as well as depletion of reduced GSH, superoxide dismustase (SOD) and catalase activity as compared to the naive group (*p* < 0.05). Treatment with *Panax quinquefolius (GIN)* (100 and 200 mg/kg) for 8 days significantly attenuated conditions of oxidative stress [alleviated MDA, nitrite concentration and restored SOD, catalase activities as well as GSH levels] as compared to control group (*p* < 0.05). Further, pretreatment with Muscimol (0.05 mg/kg) for 8 days) significantly potentiated antioxidant like profile of *Panax quinquefolius (GIN)* (100 mg/kg which was significant as compared to their effects alone in sleep deprived control animals (*p* < 0.05). However, picrotoxin (0.05 mg/kg) and flumazenil (0.5 mg/kg) pretreatment with *Panax quinquefolius (GIN)* (100 mg/kg) for 8 days significantly reversed the protective effect of *Panax quinquefolius (GIN)* in 72-h sleep deprived animals (*p* < 0.05) (Table 5) which was also significant as compared to their individual effects in sleep deprived animals.

**Table 5:** **Effect of *Panax quinquefolius (GIN)* on oxidative stress markers evaluated biochemically and its interaction with GABA modulators in 72-h sleep deprived animals**.

**S. No**	**Treatment groups (mg/kg)**	**LPO (Moles of MDA/mg protein (% of naive)**	**GSH (μmoles of GSH/ mg protein (% of naive)**	**Catalase (μmoles of H_2_O_2_/min/mg protein(% of naive)**	**Nitrite (μg/ml (% of naive)**	**SOD (Units/mg protein (% of naive)**
1	Naïve	0.204 ± 0.03 (100)	0.079 ± 0.001 (100)	0.737 ± 0.07 (100)	323.79 ± 4.58 (100)	56.71 ± 2.79 (100)
2	Control (SD-72 h)	0.728 ± 0.02^a^ (356.86)	0.0125 ± 0.002^a^ (15.82)	0.175 ± 0.0291^a^ (23.74)	720.96 ± 5.89^a^ (222.66)	12.89 ± 3.11^a^ (22.72)
3	GIN (50)	0.623 ± 0.04^a^ (305.39)	0.0203 ± 0.001^a^ (25.69)	0.292 ± 0.032^a^ (39.62)	693.56 ± 8.56^a^ (214.200)	16.32 ± 2.79^a^ (28.77)
4	GIN (100)	0.436 ± 0.010^b^^,^^c^ (213.72)	0.0449 ± 0.001^b^^,^^c^ (56.83)	0.572±0.02^b^^,^^c^ (77.61)	535.52 ± 6.39^b^^,^^c^ (165.39)	33.39 ± 1.44^b^^,^^c^ (58.87)
5	GIN (200)	0.397 ± 0.01^b^^,^^c^ (194.60)	0.0560 ± 0.003^b^^,^^c^ (70.88)	0.615 ± 0.023^b^^,^^c^ (83.44)	505.56 ± 5.40^b^^,^^c^ (156.13)	41.47 ± 2.77^b^^,^^c^ (73.12)
6	PTX (0.5)	0.734 ± 0.04^a^ (359.80)	0.0120 ± 0.003^a^ (15.18)	0.163 ± 0.02^a^ (22.11)	729.63 ± 7.58^a^ (225.34)	11.33 ± 3.14^a^ (19.97)
7	PTX (0.5) + GIN (100)	0.655 ± 0.01^a^^,^^d^ (321.07)	0.0175 ± 0.003^a^^,^^d^ (25.15)	0.227 ± 0.01^a^^,^^d^ (30.80)	705.08 ± 4.78^a^^,^^d^ (217.76)	17.44 ± 4.2^a^^,^^d^ (30.75)
8	FLU (0.5)	0.720 ± 0.02^a^ (352.94)	0.0130 ± 0.001^a^ (16.45)	0.177 ± 0.02^a^ (24.01)	722.65 ± 5.25^a^ (223.18)	13.77 ± 1.44^a^ (24.28)
9	FLU (0.5) + GIN (100)	0.679 ± 0.01^a^^,^^d^ (332.84)	0.0193 ± 0.003^a^^,^^d^ (24.43)	0.229 ± 0.03^a^^,^^d^ (31.07)	699.54 ± 4.69^a^^,^^d^ (216.04)	16.12 ± 2.11^a^^,^^d^ (28.42)
10	MUS (0.05)	0.633 ± 0.07^a^ (310.29)	0.0216 ± 0.005^a^ (27.34)	0.275 ± 0.04^a^ (37.31)	695.93 ± 5.96^a^ (214.93)	15.56 ± 2.33^a^ (27.43)
11	MUS (0.05) + GIN (100)	0.258 ± 0.01^b^^,^^d^^,^^e^ (126.47)	0.0771 ± 0.001^b^^,^^d^^,^^e^ (97.59)	0.726 ± 0.01^b^^,^^d^^,^^e^ (98.50)	362.81 ± 5.81^b^^,^^d^^,^^e^ (112.05)	50.81 ± 1.41^b^^,^^d^^,^^e^ (89.59)

*Values are expressed as mean ± SEM*.

a *p < 0.05 as compared with naive*,

b *p < 0.05 as compared with control (SD-72)*,

c *p < 0.05 as compared with GIN (50 mg/kg)*,

d *p < 0.05 as compared with GIN (100 mg/kg)*,

e *p < 0.05 as compared with MUS (0.05 mg/kg)*.

*(One-way ANOVA followed by Tukey's test). SD-72, Sleep deprivation of 72-h; GIN, Panax quinquefolius (American ginseng); PTX, Picrotoxin; FLU, Flumazenil; MUS, Muscimol*.

### Effects of *Panax quinquefolius* (GIN) and its combination with GABA modulators on sleep-deprivation induced impairment of mitochondrial respiratory enzyme complex activities

**Figure 3:**
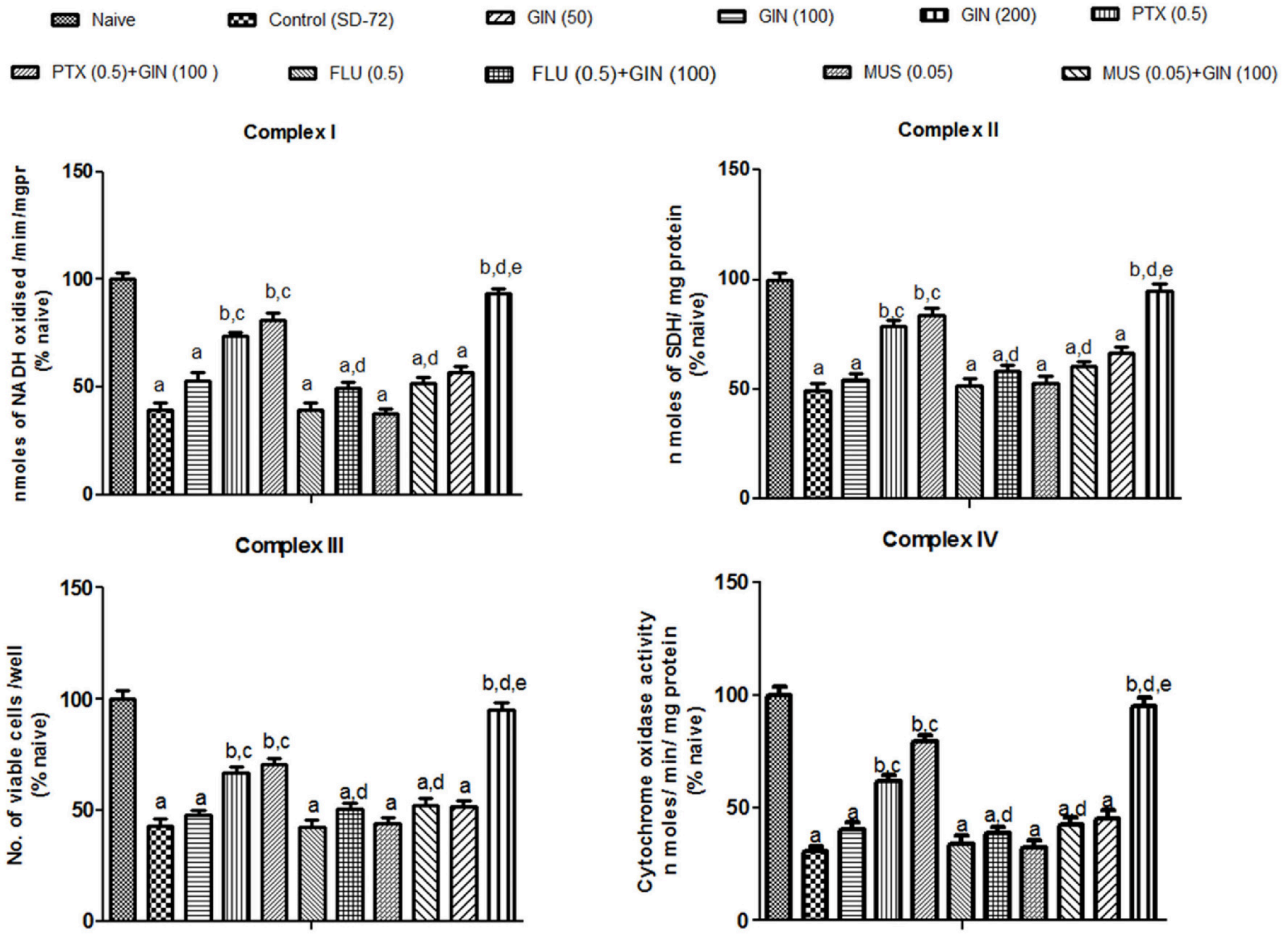
**Effects of *Panax quinquefolius* (GIN) and its combination with GABA modulators on sleep-deprivation induced impairment of mitochondrial respiratory enzyme complex activities**. Complex-I (NADH dehydrogenase activity); Complex-II (Succinate dehydrogenase activity); Complex-III (MTT/ mitochondrial redox activity); Complex IV (cytochrome c oxidase activity). Values are expressed as mean ± SEM and expressed as percentage of naive. ^a^*p* < 0.05 as compared with naive, ^b^*p* < 0.05 as compared with control (SD-72), ^c^*p* < 0.05 as compared with GIN (50 mg/kg), ^d^*p* < 0.05 as compared with GIN (100 mg/kg), ^e^*p* < 0.05 as compared with MUS (0.05 mg/kg). (One-way ANOVA followed by Tukey's test). SD-72, Sleep deprivation of 72-h; GIN:,*Panax quinquefolius* (American ginseng); PTX, Picrotoxin; FLU, Flumazenil; MUS, Muscimolss.

Mitochondrial enzyme complex activities (NADH dehydrogenase, succinate dehydrogenase and cytochrome oxidase activities as well as cell viabilities) were significantly depleted in 72-h sleep deprived animals as compared to naive group (*p* < 0.05) (Figure 3). Treatment with *Panax quinquefolius (GIN)* (100 and 200 mg/kg) significantly restored the activities of mitochondrial enzyme complexes I, II, IV and increased cell viability as compared to control (72-h SD) group (*p* < 0.05). However, *Panax quinquefolius (GIN)* (50 mg/kg) did not produce any significant restorative effect on depleted mitochondrial enzyme complex activities as compared to the 72-h sleep deprived group. Furthermore, muscimol (0.05 mg/kg) pretreatment with *Panax quinquefolius (GIN)* (100 mg/kg) for 8 days significantly potentiated their protective effect and restored mitochondrial enzyme complex activities which was significant as compared to their effects *per se* in sleep deprived animals (*p* < 0.05). However, both picrotoxin (0.05 mg/kg) and or flumazenil (0.5 mg/kg) pretreatment with *Panax quinquefolius (GIN)* (100 mg/kg) for 8 days significantly reversed its protective effect against impaired mitochondrial enzyme complex activities in 72-h sleep deprived animals (*p* < 0.05). This depicts that the restorative potential of *Panax quinquefolius (GIN)* treatment against sleep deprivation induced depleted mitochondrial enzyme complex activities was inflected by GABA modulators.

### Effect of *Panax quinquefolius* (GIN) and its modification by GABA modulators on sleep deprivation induced elevated serum corticosterone (CORT) levels

**Figure 4:**
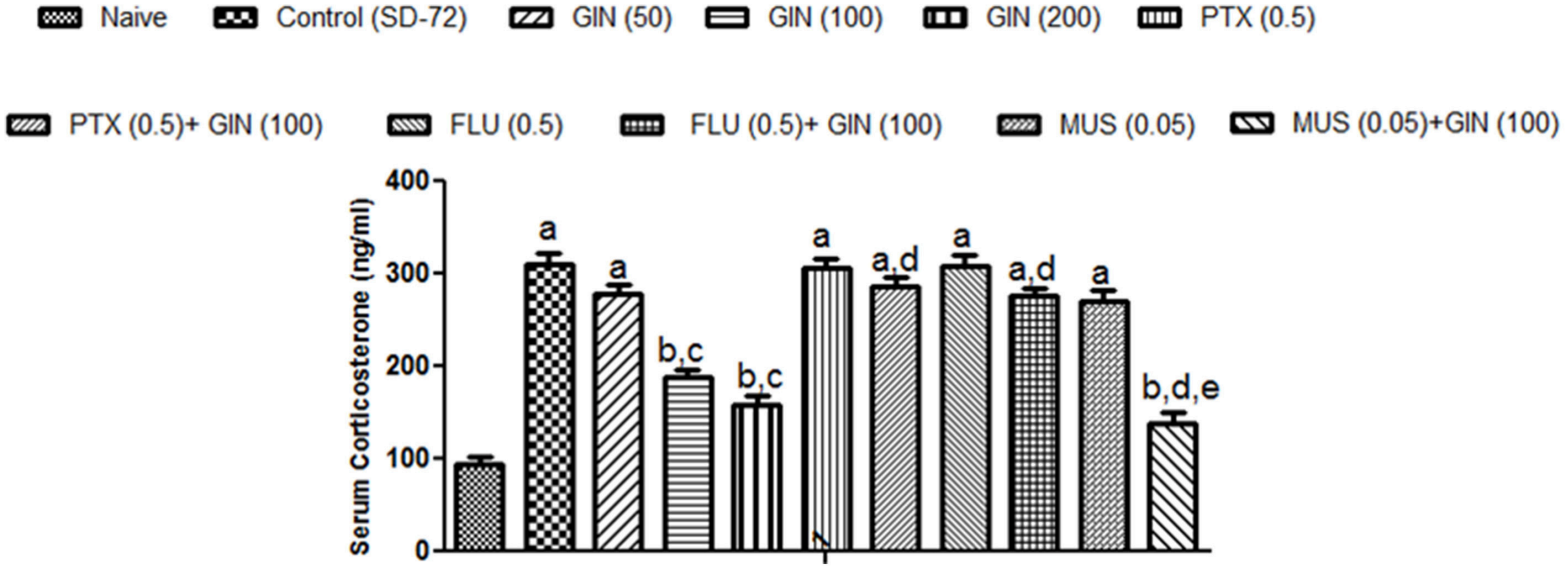
**Effect of *Panax quinquefolius* (GIN) and its modification by GABA modulators on sleep deprivation induced elevated serum corticosterone (CORT) levels**. Values are expressed as mean ± SEM. ^a^*p* < 0.05 as compared with naive, ^b^*p* < 0.05 as compared with control (SD-72), ^c^*p* < 0.05 as compared with GIN (50 mg/kg), ^d^*p* < 0.05 as compared with GIN (100 mg/kg), ^e^*p* < 0.05 as compared with MUS (0.05 mg/kg). (One-way ANOVA followed by Tukey's test). SD-72, Sleep deprivation of 72-h; GIN, *Panax quinquefolius* (American ginseng); PTX, Picrotoxin; FLU, Flumazenil; MUS, Muscimol.

Sleep deprived (72-h) animals showed a significant increase in serum CORT levels as compared with naïve group (*p* < 0.05). Treatment with *Panax quinquefolius (GIN)* (100, 200 mg/kg) significantly (*p* < 0.05) attenuated serum CORT levels as compared with control group (72-h SD) (Figure 4). Treatment with *Panax quinquefolius (GIN)* (50 mg/kg) did not produce any significant restorative effect on serum CORT levels as compared to the 72-h sleep deprived group. The protective effect of *Panax quinquefolius (GIN)* treatment against elevated serum CORT levels was found to be regulated by GABA modulators pretreatment. 8 days picrotoxin (0.05 mg/kg) and or flumazenil (0.5 mg/kg) pretreatment with *Panax quinquefolius (GIN)* (100 mg/kg) significantly reversed this protective effect of *Panax quinquefolius (GIN)* (*p* < 0.05). Furthermore, in 72-h SD animals pretreatmemnt of muscimol (0.05 mg/kg) with *Panax quinquefolius (GIN)* (100 mg/kg) significantly (*p* < 0.05) potentiated their protective effect (depleted serum CORT level), as compared with their individual effects alone.

### Effect of *Panax quinquefolius* (GIN) and its modification by GABA modulators on TNF-α in sleep-deprived mice

**Figure 5:**
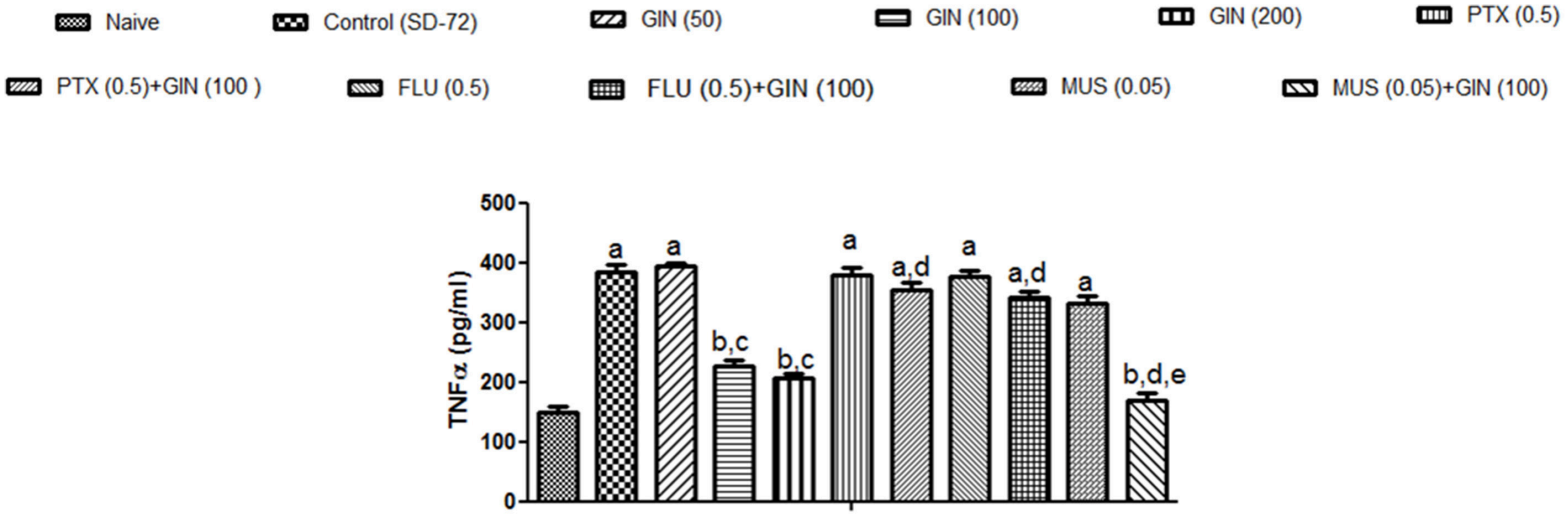
**Effect of *Panax quinquefolius* (GIN) and its modification by GABA modulators on TNF-α in sleep-deprived mice**. Values are expressed as mean ± SEM. ^a^*p* < 0.05 as compared with naive, ^b^*p* < 0.05 as compared with control (SD-72), ^c^*p* < 0.05 as compared with GIN (50 mg/kg), ^d^*p* < 0.05 as compared with GIN (100 mg/kg), ^e^*p* < 0.05 as compared with MUS (0.05 mg/kg). (One-way ANOVA followed by Tukey's test). SD-72, Sleep deprivation of 72-h; GIN, *Panax quinquefolius* (American ginseng); PTX, Picrotoxin; FLU, Flumazenil; MUS, Muscimol.

As depicted in Figure 5, 72-h sleep deprivation significantly increased encephalic TNF-α levels as compared to naïve group (*p* < 0.05). Unlike with lower dose (50 mg/kg), treatment with *Panax quinquefolius (GIN)* (100, 200 mg/kg) for 8 days significantly attenuated TNF α levels as compared to control group (*p* < 0.05). Furthermore, picrotoxin (0.5 mg/kg) and flumazenil (0.5 mg/kg) pretreatment with *Panax quinquefolius (GIN)* (100 mg/kg) significantly reversed this protective effect of *Panax quinquefolius (GIN)* (raised TNF-α level) in 72-h sleep deprived animals (*p* < 0.05). However, muscimol (0.05 mg/kg) pre-treatment with *Panax quinquefolius (GIN)* (100 mg/kg) potentiated their protective effect (attenuated TNF-α levels) which was significant as compared to their effect alone in 72-h sleep deprived animals (*p* < 0.05).

### Effect of *Panax quinquefolius* (GIN) and its modification by GABA modulators on neuronal morphology as depicted by histopathological sections of sleep deprived mice brains

**Figure 6:**
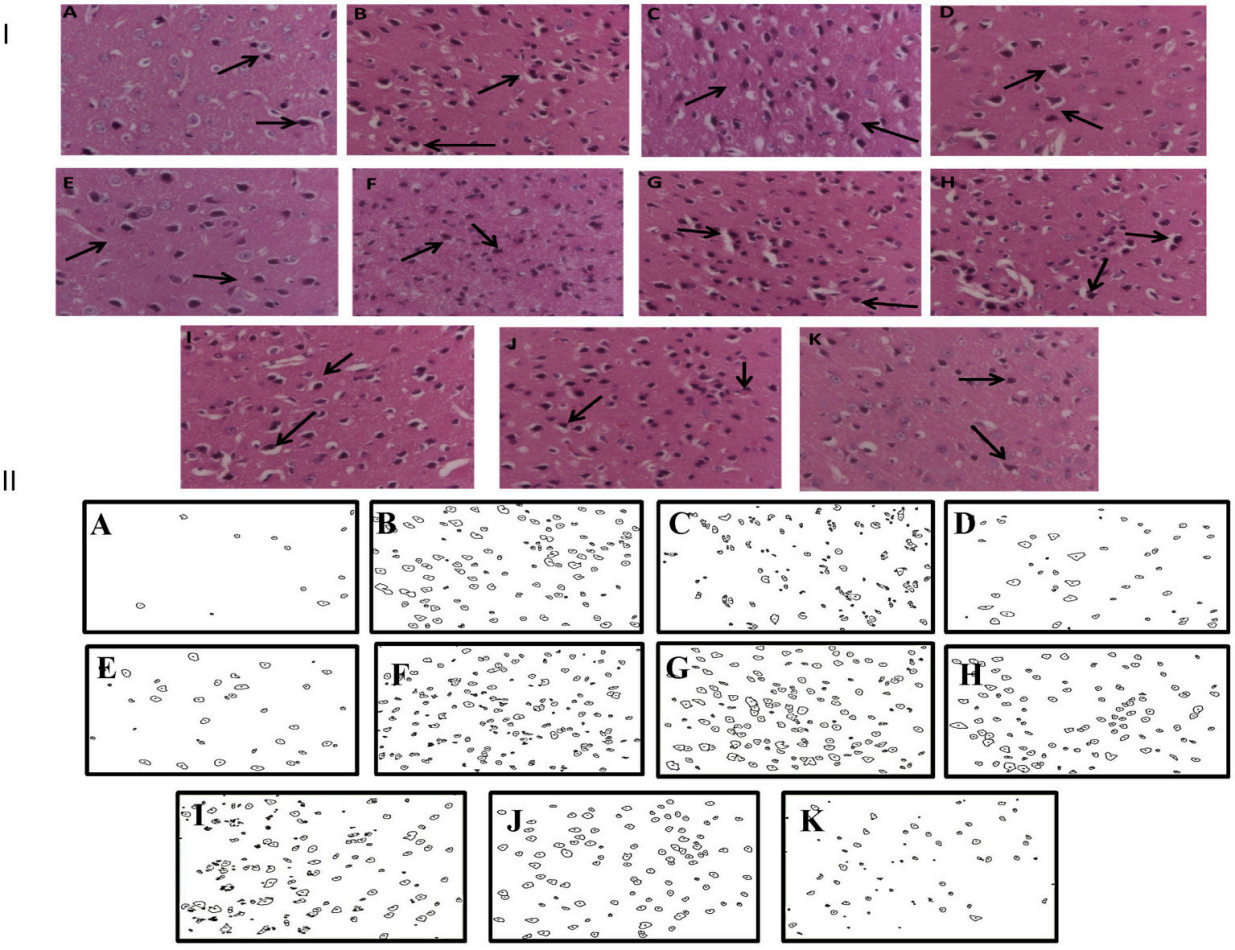
**(I)** Effects of *Panax quinquefolius* (GIN) alone and in combination with GABA modulators on neuronal morphology as depicted by photomicrographs of histopathological sections (at 40X) of sleep deprived mice brains. **(A)** Naïve; **(B)** Sleep deprived (72 h); **(C)** GIN (50); **(D)** GIN (100); **(E)** GIN (200); **(F)** PTX (0.5); **(G)** PTX (0.5) + GIN (100); **(H)** FLU (0.5); **(I)** FLU (0.5) + GIN (100); **(J)** MUS (0.05); **(K)** MUS (0.05) + GIN (100). 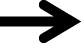:Pyknotic cells depicting mild inflammation of neurons **(II)** The histopathological sections as modified by software for processing; showing outliners of pyknotic cells; **(A)** Naïve; **(B)** Sleep deprived (72 h); **(C)** GIN (50); **(D)** GIN (100); **(E)** GIN (200); **(F)** PTX (0.5); **(G)** PTX (0.5) + GIN (100); **(H)** FLU (0.5); **(I)** FLU (0.5) + GIN (100); **(J)** MUS (0.05); **(K)** MUS (0.05) + GIN (100).

Histopathological evaluation of *thalamo-cortical region* of brains of sleep deprived mice was conducted by light microscopy. Representative images of brain sections from different animal groups are shown in Figure 6I. In the histopathological evaluation, brains of naïve animals showed comparatively undamaged and optimum sized neuronal cells and cell layers as compared to control, i.e., 72-h sleep deprived animals. However, disorganization of various cell layers, increase in density of pyknotic cells (signifying neuroinflammatory changes), was observed in thalamo-cortical regions of the brains of sleep deprived animals. Treatment with *Panax quinquefolius (GIN)* (100 and 200 mg/kg) attenuated these neuroinflammatory changes in neuronal morphologies in sleep deprived animals as compared to the control group (Figure 6I). In addition, the number of pyknotic cells, per 128,800 square pixels of area were found to be higher in the thalamo-cortical regions of 72-h sleep deprived animals (control group), as compared to the naïve group (*p* < 0.05). 8 days treatment with *Panax quinquefolius (GIN)* 100 and 200 mg/kg significantly brought about a reduction in number of pyknotic cells as compared to the control group (*p* < 0.05). Furthermore, this protective effect of *Panax quinquefolius (GIN)* treatment against increased pyknotic cell density was significantly regulated by GABA modulators pretreatments. While, picrotoxin (0.5 mg/kg) and or flumazenil (0.5 mg/kg) pretreatment with *Panax quinquefolius (GIN)* (100 mg/kg) significantly reversed the protective effect of *Panax quinquefolius (GIN)*. Further, pretreatment with muscimol (0.05 mg/kg) significantly potentiated its *(GIN)* protective effect against (decreased pyknotic cell density), which was significant as compared to their effect *per se* (*p* < 0.05) (Figures 6II, 7).

**Figure 7:**
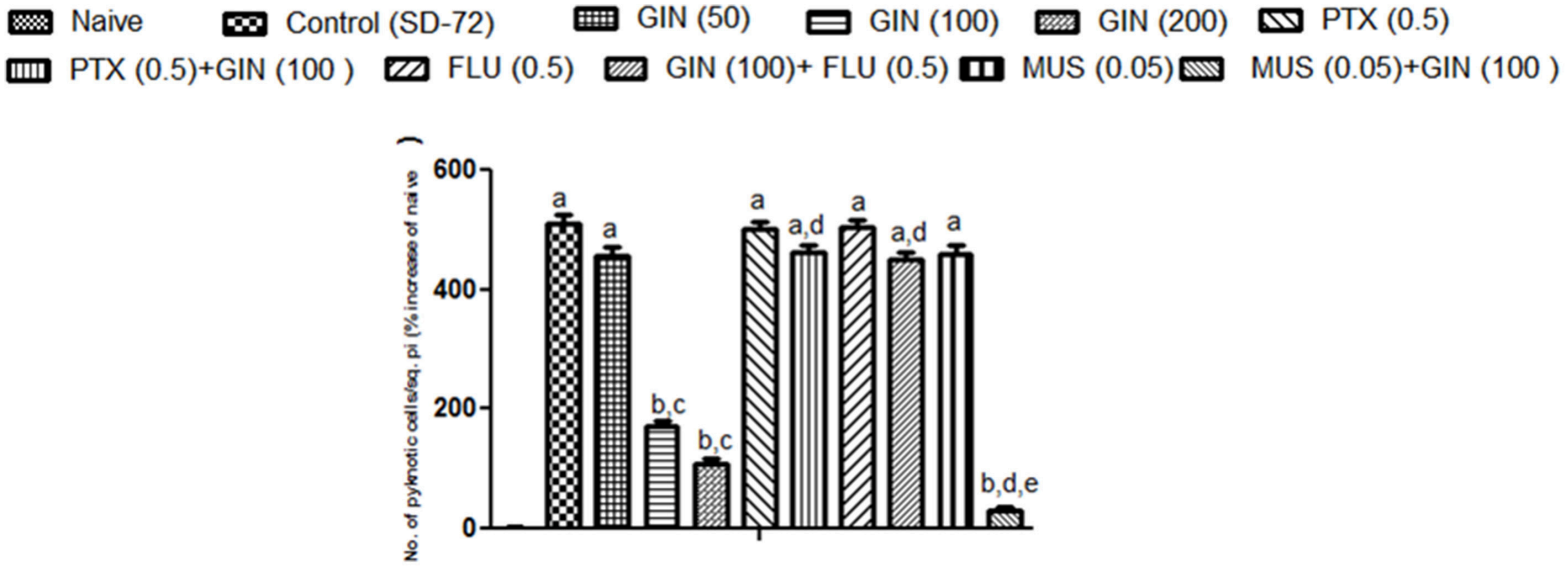
**Graph representing the effect of *Panax quinquefolius* (GIN) and modulatory effect of GABA modulators on ginseng's activity on pyknotic cell expression per 1228800 square pixel; Values are expressed as mean ± SEM and expressed as percentage increase of naive**. ^a^*p* < 0.05 as compared with naive, ^b^*p* < 0.05 as compared with control (SD-72), ^c^*p* < 0.05 as compared with GIN (50 mg/kg), ^d^*p* < 0.05 as compared with GIN (100 mg/kg), ^e^*p* < 0.05 as compared with MUS (0.05 mg/kg). (One-way ANOVA followed by Tukey's test). SD-72, Sleep deprivation of 72-h; GIN, *Panax quinquefolius* (American ginseng); PTX, Picrotoxin; FLU, Flumazenil; MUS, Muscimol.

## Discussion

The findings of the present study encompass the behavioral, biochemical as well as neuroinflammatory alterations accumulated as a result of 72-h of SD as well as the neuroprotective effect and underlying mechanisms of Panax quinquefolius against the above mentioned manifestations. Sleep is an important homeostatic phenomenon and SD has been often linked to several neurological debilities for ex. depression, anxiety like behavior, cognitive dysfunction, hyperalgesic conditions etc. Several mechanisms have been suggested to explain the complex aetiological cascade responsible for development of SD induced neurological comorbidities particularly oxidative stress, mitochondrial dysfunction, HPA-axis activation, neuroinflammation as well as apoptosis (Kumar and Chanana, 2014).

In our study, subjection of animals to 72-h of SD brought about intense behavioral alterations like motor dysfunction and anxiety like behavior as evidenced by decreased locomotor activity as well as increased latency and decreased number of entries as well as duration in the mirror cube in mirror chamber test paradigm, as compared to the naïve group. Additionally, biochemical correlates in the present study signify the accumulation of oxidative free radicals (characterized by increased lipid peroxidation, nitric oxide load and depleted reduced glutathione, catalase and SOD activities) and depleted activity of mitochondrial enzyme complexes (NADH dehydrogenase, succinate dehydrogenase activity, cytochrome C oxidase and impaired the neural cell viability) as a direct consequence of 72-h of SD. Distinct lines of evidence suggest that SD on chronic basis may lead to differential alterations in the GABA mediated inhibitory signaling and may also precipitate dynamic changes in the density of GABAA receptors [GABA_A_R] in various areas of the brain responsible for regulation of sleep/ wake flip flop cycle (Watt et al., 2000; Tononi and Cirelli, 2006). It can be purported that conditions of oxidative stress that may be precipitated via interplay between NOX2 activation and mitochondrial enzyme complex impairment (as a consequence of SD) may prove detrimental to GABA-ergic neurons as well as inhibitory signaling. Studies have demonstrated a direct link between NOX2 upregulation and depleted GABA ergic inhibitory signaling. It has been evident that a paracrine effect of increased NOX2 activity is that it causes dysfunction of the fast spiking, parvalbumin (PV)-expressing subpopulation of inhibitory GABAergic interneurons, thereby blocking the inhibitory effect (Behrens et al., 2007; Dugan et al., 2009). Further, this effect of NOX2 has been reported to be regulated via proinflammatory cytokines like IL-6 (Behrens et al., 2008) levels. Thus, with the above backdrop it can be concluded that GABA-ergic downregulation may serve as pivotal aetiological links in development of secondary neurological disorders associated with SD like motor dysfunctions, anxiety like behavior etc. (Kumar and Singh, 2009).

SD has been documented to aid microglial activation and thus lead to elevated levels of pro inflammatory markers such as tumor necrosis factor-α (TNF-α), interleukin-6 (IL-6), and C-reactive protein (CRP) (Hu et al., 2003; Meier-Ewert et al., 2004). In consistence with the above reports, the current study too demonstrated a significant increase in the encephalic levels of TNF-α in sleep deprived animals as compared to the naive animals, indicating the role of neuroinflammation in SD induced pathophysiologies. This may signify a situation of microglial activation as a consequence of SD. Recently, TNF-α levels have also shown to regulate GABA ergic inhibitory synapse function. The activation of TNF-α receptor 1 (TNFR1), may lead to attenuation of inhibitory synaptic strength as well as endocytosis of membranal GABAARs (Stellwagen et al., 2005; Pribiag and Stellwagen, 2013). Thus, through the present study it can be purported that one of the mechanisms behind SD mediated alterations in GABA-ergic signaling and consequent development of secondary neurological debilities could be mediated via TNFR1 activation.

Being a kind of stress, exposure to sleep deprivation may induce activation of the hypothalamo-pituitary-adrenal (HPA) axis (Shansky and Lipps, 2013). This HPA axis activation provokes corticosterone secretion which further triggers the release of oxidative stress markers causing impairment of various neurological functions. In concordance with the above mentioned reports, the present study too demonstrated that exposure of animals to SD of 72-h led to HPA-axis activation demonstrated by considerably raised serum corticosterone levels in comparison to the naïve animals. Supplementary to this the increased corticosterone levels have been documented to alter GABA ergic signaling. This is induced via alterations in receptor configurations, decrease in GABA release from presynaptic neurons as well as change in the levels of GAD 65 (glutamate decarboxylase employed in the synthesis of GABA) (Verkuyl et al., 2005; Lussier et al., 2013). This may suggest another plausible mechanism for SD induced damage to the GABA-ergic signaling and consequent precipitation of anxiety like behavior.

The current study targeted the thalamo-cortical regions of the brain for studying the histopathological correlates in brains of SD animals because synchronization and desynchronization of thalamo-cortical circuits have been found to play an important role in the electrophysiological transitory events between sleep and wakefulness (Steriade, 1996; Pace-Schott and Hobson, 2002). Histopathological evaluations in the present study demonstrated neuroinflammatory responses and apoptotic conditions in discrete brain areas of sleep deprived animals as manifested by significantly higher density of pyknotic neuronal cells when compared to naïve animals. This may be responsible for disruptive functioning of the thalamo-cortical circuitry as well as disruption of sleep wake homeostasis which may further serve as causative for development of anxiety like behavior in SD animals.

Several studies have reported that the CNS benefits of *Panax quinquefolius (GIN)* can be attributed to the pharmacological activities of its active constituents, the triterpenoid saponins called ginsenosides. These ginsenosides influence neurological functions in terms of its anti-inflammatory, antioxidant, and antiapoptotic like properties (Leung and Wong, 2010; Radad et al., 2011). In concordance with the above mentioned research inferences, the present study too reported that *Panax quinquefolius (GIN)* treatment for 8 days in sleep deprived animals significantly attenuated the anxiogenic like behavior, oxidative stress, mitochondrial dysfunction, serum corticosterone and pro-inflammatory marker (TNF-α) levels as well as minimized histological neuronal changes as compared to the sleep deprived vehicle treated animals. Although GABA is known to be a primarily involved in consequences of SD, but sleep research in recent times has also pointed toward significant alterations in 5-HT neurotransmission as well as neurochemical metabolism (under the control of molecules like 5-hydroxytryptamine transporter, 5-HTT and Glycogen synthase kinase 3-β) as a direct consequence of SD (Benedetti et al., 1999, 2012; Monti et al., 2011; Zant et al., 2011; Elmenhorst et al., 2012; Sharma et al., 2015). Also, 5-HT receptor antagonists have recently be classified for their promising role against SD-induced brain dysfunctions (Youngblood et al., 1999; Morairty et al., 2008) affirming that the secondary neurological debilities manifested as a direct concern of SD can also be accredited to altered 5-HT neurotransmission. Also the active constituents of *Panax quinquefolius* (ginsenosides, other phytosterols as well as total saponins) have shown to be substantially involved in modulation of 5-HT neurotransmission as well expression and modulation of receptor activity. It has been demonstrated that protective effect of *Panax quinquefolius* against neuropsychiatric disorders was manifested through its specific effects on serotonin levels in specific brain areas (Beveridge et al., 2002; Chatterjee et al., 2012). Furthermore, ginsenoside Rg and ginsenoside metabolites such as compound K and M4 have reported to cause inhibition of 5-HT_3_ receptor-gated ion currents in Xenopus oocytes expressing 5-HT_3_A receptors (Choi S. et al., 2003; Lee et al., 2004). Lee et al mechanistically depicted the effect of ginsenosides on 5-HT receptors via the interaction with residues F292, V291, and I295 in the channel gating region of transmembrane domain 2 (TM2) of the receptor (Lee et al., 2004). Therein following the above background the potential neuroprotective effect of *Panax quinquefolius* against SD induced anxiety like behavior can also be attributed to alteration of 5-HT neurotransmission.

Furthermore, it was hypothesized that neuroproprotective potential of *Panax quinquefolius's (GIN)* in SD induced anxiety like behavior could be manifested by its GABA modulating actions. Therefore, our study undertook an extended effort to see interaction of *Panax quinquefolius (GIN)* with GABA-ergic modulators. In the present study, picrotoxin and flumazenil (GABA Cl^−^ channel inhibitor as well as GABA-benzodiazepine receptor inhibitor respectively) pre-treatment with *Panax quinquefolius (GIN)* for 8 days significantly reversed the protective effect (depleted gross motor activity, increased anxiety like behavior, oxidative stress, mitochondrial dysfunction, elevated corticosterone and TNF-α levels) of *Panax quinquefolius (GIN)* with respect to its action alone in animals subjected to SD. However, muscimol (GABA A selective agonist) pre-treatment with *Panax quinquefolius (GIN)* significantly potentiated its neuroprotective effect suggesting the involvement of GABA-ergic modulating mechanisms underlying the neuroprotective effect of *Panax quinquefolius (GIN)* against SD induced anxiety like behavior, oxidative stress and neuroinflammation. The GABA ergic modulatory potential of *Panax quinquefolius* as purported by the present study is line with earlier mentioned reports where *Panax quinquefolius (GIN)* has shown to affect the neurotransmission of acetyl choline and GABA by mechanisms involving the regulation of the expression of the synthetic enzyme, regulation of the ligand binding capacities, neurotransmitter release as well as signaling pathways involved in the particular neurotransmitter system (Yuan et al., 1998; Kim et al., 2001; Choi S. et al., 2003; Lee et al., 2013).

**Figure 8:**
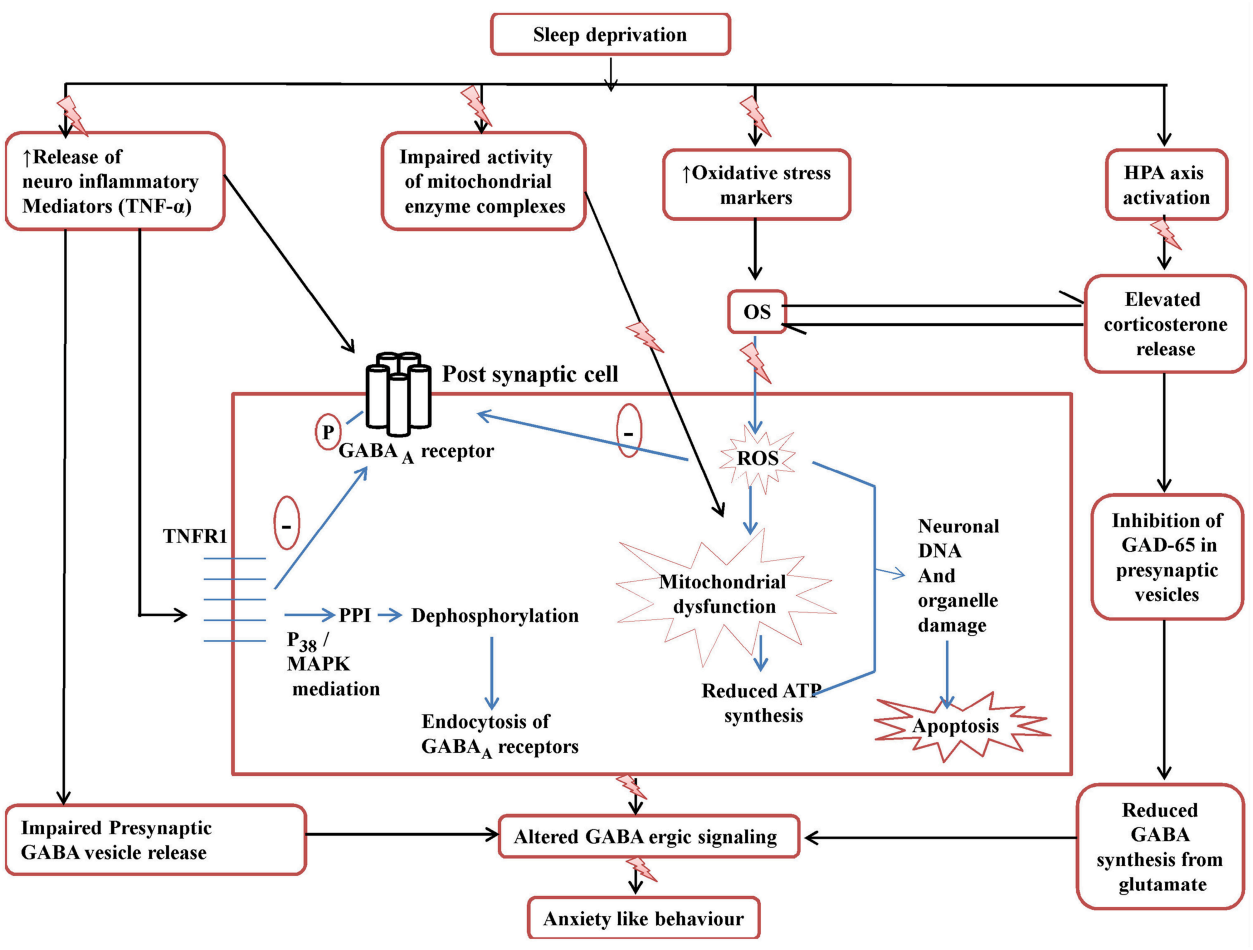
**Posssible GABA-ergic involvement in the development of SD induced anxiety like behavior and targets in the neuroprotective effect of *Panax quinquefolius* (American ginseng)**. ROS, reactive oxidative species; OS, oxidative stress; GAD65, glutamate decarboxylase; MAPK, Mitogen activated protein kinase; PPI, protein phosphatase I; TNFR1, TNF-α receptor 1. The figure clearly depicts the various mechanisms involved in disruptive GABA-ergic inhibitory signaling precipitated by sleep deprivation which may lead to development of anxiety like behavior. The elevated TNF-α levels affect GABA-ergic signaling by decreasing the release of GABA vesicles presynaptically as well as by causing endocytosis of post-synaptic GABAA receptors, mediated by its binding to TNFR1. The activation of TNFR1 dephosphorylates GABAA receptors via PPIvia mediation of p38 and MAPK pathways and thus causes endocytosis of GABAA. Also the elevated corticosterone levels lead to decreased activity of GAD65 the main enzyme involved in the synthesis of GABA from glutamate, in the presynaptic cell. All these events contribute to disruptive GABA-ergic inhibitory signaling.

But still the results of the present study would require robust cellular confirmations giving a proposition that further research in this area may be required. Figure 8 tries to explain the findings of the present study and the plausible targets of action of *Panax quinquefolius (GIN)* against SD induced anxiety like behavior.

## Conclusion

The present study recommended the possible involvement of GABA-ergic mechanism in the protective effect of *Panax quinquefolius (GIN)* against 72-h sleep deprivation induced anxiety like behavior, oxidative stress, mitochondrial dysfunction and neuroinflammation. Further, study provides a hope that *Panax quinquefolius (GIN)* could be used as an adjuvant therapy in the management of SD induced anxiety like behavior.

## Author contributions

PC contributed to the study design, performed the research, as well as worked upon data and statistical analysis. Prof. AK contributed in design of study as well as critical analysis of the manuscript.

## Funding

The present work was financially supported by University Grants Commission via Research Fellowship in Sciences for Meritorious Students, UGC- RFSMS [award number: F.5-94/2007(BSR)] awarded to Ms. PC. It is further declared that the funding body played no role in manuscript writing, study design, interpretation of data, or in decision to submit the article to the journal. All above mentioned steps have been performed by the authors.

### Conflict of interest statement

The authors declare that the research was conducted in the absence of any commercial or financial relationships that could be construed as a potential conflict of interest.

## Abbreviations

BF: Basal forebrain
CRP: C-Reactive Protein
DNA: deoxyribonucleic acid
GABA: gamma amino butyric acid
NO: nitric oxide
GSH: reduced glutathione
H_2_O_2_: hydrogen peroxide
MDA: malondialdehyde
SD, Sleep Deprivation, TNF: tumor necrosis factor
VLPO: Venterolateral preoptic.
